# Applications of amyloid, tau, and neuroinflammation PET imaging to Alzheimer's disease and mild cognitive impairment

**DOI:** 10.1002/hbm.24782

**Published:** 2019-09-14

**Authors:** Avinash Chandra, Polytimi‐Eleni Valkimadi, Gennaro Pagano, Oliver Cousins, George Dervenoulas, Marios Politis, for the Alzheimer's Disease Neuroimaging Initiative

**Affiliations:** ^1^ Neurodegeneration Imaging Group (NIG), Institute of Psychiatry, Psychology and Neuroscience (IoPPN) King's College London (KCL) London UK

**Keywords:** Alzheimer's disease, amyloid, mild cognitive impairment, neuroinflammation, neuropathology, PET, tau

## Abstract

Alzheimer's disease (AD) is a devastating and progressive neurodegenerative disease for which there is no cure. Mild cognitive impairment (MCI) is considered a prodromal stage of the disease. Molecular imaging with positron emission tomography (PET) allows for the *in vivo* visualisation and tracking of pathophysiological changes in AD and MCI. PET is a very promising methodology for differential diagnosis and novel targets of PET imaging might also serve as biomarkers for disease‐modifying therapeutic interventions. This review provides an overview of the current status and applications of *in vivo* molecular imaging of AD pathology, specifically amyloid, tau, and microglial activation. PET imaging studies were included and evaluated as potential biomarkers and for monitoring disease progression. Although the majority of radiotracers showed the ability to discriminate AD and MCI patients from healthy controls, they had various limitations that prevent the recommendation of a single technique or tracer as an optimal biomarker. Newer research examining amyloid, tau, and microglial PET imaging in combination suggest an alternative approach in studying the disease process.

## 1 INTRODUCTION

Alzheimer's disease (AD) is the most common cause of dementia worldwide. It is estimated that by 2050, 1 in 85 people worldwide will develop AD (Brookmeyer, Johnson, Ziegler‐Graham, & Arrighi, 2007). The prodromal phase of AD, defined as mild cognitive impairment (MCI), is characterised by declines in performance of one or more cognitive domains with the preservation of functional independence (Petersen, 2004). Central nervous system (CNS) degeneration and disease neuropathology predates AD and MCI. This is particularly true in presymptomatic carriers of apolipoprotein E (*APOE*) ε4 (Reiman et al., 2009), which is the leading genetic risk factor for AD (Liu et al., 2014). The hallmark neuropathological substrates for AD and MCI are β‐amyloid (Aβ) plaques and intracellular tau neurofibrillary tangles (NFTs). One major theory, the ‘amyloid cascade hypothesis’, suggests that the overproduction combined with dysfunctional clearance of Aβ is the fundamental event that initiates AD pathogenesis (Hardy & Selkoe, 2002). However, several lines of evidence challenge this assumption. For example, approximately 30% of healthy elderly individuals have significant levels of Aβ deposition without apparent clinical symptoms (Rowe et al., 2010). In AD, histopathological evidence suggests that Aβ levels are a poor predictor of severity of cognitive impairment (Giannakopoulos et al., 2003) and anti‐amyloid interventions have demonstrated limited efficacy in clinical trials (Karran & Hardy, 2014). Presence and extent of hyperphosphorylated tau‐based NFT pathology is positively associated with disease duration and severity of cognitive symptoms (Gómez‐Isla et al., 1997). In addition to the more traditional markers of AD pathology, the existence of neuroinflammation in AD is currently well established. Whilst the initial inflammatory response aims to ameliorate neuronal injury, abnormally prolonged microglial activation can have detrimental effects and potentially serve to exacerbate neurodegeneration (Fakhoury, 2018).

Positron emission tomography (PET) is a neuroimaging tool designed to measure *in vivo* molecular processes in the brain. PET radioligands bind a target, such as a receptor, a transporter, or an enzyme. Degree of tracer binding or uptake is used to quantify neuropathology. This technology may be particularly useful for diagnostic purposes, treatment planning, and to assess disease progression in neurological illnesses (Politis & Piccini, 2012). PET biomarkers that have been recommended to improve diagnostic accuracy for AD and MCI include decreased cerebral metabolism on [^18^F]fludeoxyglucose (FDG) PET and increased Aβ deposition on amyloid PET (Albert et al., 2011; McKhann et al., 2011). Rates of cerebral metabolism do not explicitly elucidate potential disease‐causing neuropathology, but more likely highlight the degree of neuronal activity (Marcus, Mena, & Subramaniam, 2014). Characteristic region‐based patterns of brain hypometabolism have been established within a range of different dementia aetiologies (Kato, Inui, Nakamura, & Ito, 2016; Minoshima, Frey, Koeppe, Foster, & Kuhl, 1995; Moonga et al., 2017; Mosconi et al., 2008; Nestor, Caine, Fryer, Clarke, & Hodges, 2003). PET tracers that measure Aβ burden, tau aggregation, and neuroinflammation provide remarkable insight directly into the processes underlying the pathophysiology of AD and MCI; however, the latter two have not yet been suggested for use in clinical practice. This review describes the recent developments and current applications of PET imaging of amyloid, tau, and neuroinflammation in AD and MCI. Abnormalities on magnetic resonance imaging (MRI) have also been used as clinically relevant imaging markers for AD and MCI (Chandra, Dervenoulas, Politis, & Alzheimer's Disease Neuroimaging Initiative, 2018); however, this is not within the scope of the current work.

## 2 AMYLOID STUDIES

### 2.1 ^11^C‐labelled amyloid tracers

Klunk et al. discovered what would become the most heavily researched amyloid PET radiotracer, ^11^C‐labelled Pittsburgh compound B ([^11^C]PiB; Table 1) (Klunk et al., 2004). In their seminal study, it was shown that this benzothiazole‐based radioligand was capable of discriminating between patients with a diagnosis of extremely mild AD and healthy controls. Areas such as frontal and temporoparietal cortex, which are particularly susceptible to Aβ pathology, demonstrated increased [^11^C]PiB retention in AD. These findings have been replicated, and moreover, recent findings highlight intermediate regional binding of [^11^C]PiB in MCI when compared to healthy controls and AD patients (Rowe et al., 2010; Villemagne et al., 2013). PiB PET has demonstrated the ability to bind to cored and neuritic Aβ plaques, and also diffuse ones (Ikonomovic et al., 2008).

**Table 1:** Studies examining *in vivo* regional brain uptake using amyloid tracers in AD and MCI

Study	Amyloid tracer	Study population	Main findings
Klunk et al. (2004)	[^11^C]PiB	16 AD patients, 9 healthy controls	Compared to healthy controls, AD patients showed increased tracer retention in all four lobes of the cortex, in addition to the striatum. Regions including pons, subcortical white matter, and the cerebellum, which are typically unaffected by amyloid depositions showed no such differences. Regional amyloid retention was negatively associated with glucose metabolism as measured by FDG PET.
Rowe et al. (2010)	[^11^C]PiB	53 AD patients, 57 MCI patients, 177 healthy controls	Relative to healthy controls, AD and MCI patients demonstrated increased PiB binding in the cortex. This was higher for AD patients compared to those with MCI. Regions that showed notable tracer uptake for patients included the precuneus, posterior cingulate, orbitofrontal, lateral temporal cortex, and striatum.
Brück et al. (2013)	[^11^C]PiB	29 MCI patients	Elevated amyloid retention in areas within the cingulate and frontal and temporal cortex was predictive of conversion from MCI to AD.
Wolk et al. (2012)	[^11^C]PiB [^18^F]florbetapir	12 AD patients, 14 cognitively normal subjects	Both amyloid tracers showed that AD patients had higher binding in areas that included the anterior cingulate, posterior cingulate, precuneus, frontal, parietal, and lateral temporal cortex compared to cognitively normal subjects. There was also region‐based positive correlations between the two ligands.
Camus et al. (2012)	[^18^F]florbetapir	13 AD patients, 12 MCI patients, 21 healthy controls	In AD, increased tracer retention was demonstrated for all 4 lobes of the cortex, in addition to the precuneus and cingulate cortex when compared to healthy controls. MCI patients demonstrated a similar pattern, but primarily in the posterior cingulate cortex.
Johnson et al. (2013)	[^18^F]florbetapir	45 AD patients, 60 MCI patients, 79 healthy controls	Amyloid retention was higher in frontal, temporal, and parietal cortical areas, in addition to the cingulate and precuneus for AD patients compared to healthy controls. Patients with MCI showed similar but intermediate effects.
Namiki et al. (2015)	[^18^F]florbetapir	15 AD patients, 15 MCI patients, 18 cognitively normal subjects	AD patients showed increased [^18^F]florbetapir binding in the frontal cortex, temporal cortex, parietal cortex, cingulate cortex, and precuneus compared to cognitively normal subjects. No such differences were found for MCI patients relative to controls. Higher binding in the temporal, parietal, and cingulate cortex was found in AD versus MCI.
Wong et al. (2010)	[^18^F]florbetapir	16 AD patients, 16 healthy controls	Compared to healthy controls, AD patients displayed greater tracer retention in frontal, temporal, occipital, parietal, cingulate cortices, in addition to the precuneus.
Barthel et al. (2011)	[^18^F]florbetaben	81 AD patients, 69 healthy controls	Cortical uptake in all regions including temporal, parietal, frontal, occipital, and both posterior and anterior cingulate cortex was higher in AD patients when compared to healthy controls. The posterior cingulate cortex had the best ability to discriminate between the two groups. Regional uptake values had a sensitivity of 85% and a specificity of 91% and were inversely related to global cognitive and memory performance.
Nelissen et al. (2009)	[^18^F]flutemetamol	Eight AD patients, eight healthy controls	Regions that showed increased tracer retention for AD patients included the anterior cingulate, frontal cortex, lateral temporal cortex, parietal cortex, posterior cingulate, sensorimotor cortex, and striatum relative to healthy controls.
Rowe et al. (2008)	[^18^F]florbetaben	15 AD patients, 15 healthy controls	Regional binding was observed in cortical areas for AD patients, moreso in the frontal cortex, precuneus and posterior cingulate than in parietal and lateral temporal areas compared to controls. Limited binding was observed in occipital, sensorimotor, and mesial temporal areas.
Villemagne et al. (2011)	[^18^F]florbetaben	30 AD patients, 20 MCI patients	Compared to controls, increased florbetaben binding was seen in prefrontal, orbitofrontal, cingulate, parietal, occipital, and temporal cortical areas, in addition to the putamen, thalamus, and caudate nuclei. A similar pattern was shown for MCI in the ventrolateral prefrontal, orbitofrontal, posterior cingulate, parietal, lateral temporal cortex, and the putamen.
Vandenberghe et al. (2010)	[^11^C]PiB [^18^F]flutemetamol	27 AD patients, 20 aMCI patients, 15 healthy controls	Tracer uptake in cortical areas including lateral frontal, lateral temporal, medial temporal, occipital, lateral parietal, and cingulate, in addition to the striatum, but not the pons and subcortical white matter, differentiated AD patients and controls. Regional correlations were also shown with [^18^F]flutemetamol and PiB uptake.
Villemagne et al. (2012)	[^11^C]PiB [^18^F]florbetaben	10 AD patients, 10 healthy controls	For AD patients, both tracers demonstrated significantly higher uptake compared to controls in the prefrontal, orbitofrontal, gyrus rectus, cingulate, parietal, lateral occipital, temporal cortex, in addition to the caudate and putamen. However, uptake was shown in the thalamus for [^11^C]PiB but not [^18^F]flutemetamol.
Lowe et al. (2017)	[^11^C]PiB [^18^F]flutemetamol	21 AD patients, 30 young cognitively normal subjects, 31 elderly cognitively normal subjects	In the cingulate, caudate, precuneus, insula, medial temporal cortex, occipital cortex, orbitofrontal cortex, pallidum, paracentral lobule, parietal cortex, postcentral cortex, precentral cortex, prefrontal cortex, primary visual cortex, putamen, rolandic operculum, supplementary motor area, and thalamus higher tracer retention for both [^18^F]flutemetamol and [^11^C]PiB was found in AD patients compared to controls.

Abbreviations: AD, Alzheimer's disease; aMCI, amnestic mild cognitive impairment; FDG, [^18^F]fludeoxyglucose; MCI, mild cognitive impairment; PET, positron emission tomography.

Amyloid load as measured by [^11^C]PiB *in vivo* has also been associated with other markers of AD including brain atrophy (Archer et al., 2006), medial temporal hypometabolism on [^18^F]FDG PET, impaired memory performance (Frings, Spehl, Weber, Hüll, & Meyer, 2013), and Aβ pathology from post‐mortem brain tissue (Driscoll et al., 2012). Regarding disease progression, cortical [^11^C]PiB uptake in frontal, temporal, and cingulate areas was found to be predictive of phenoconversion from MCI to AD (Brück et al., 2013). It is important to concede that MCI patients display a bimodal distribution for amyloid uptake where amyloid load is significantly elevated in a proportion of some MCI patients but not others (Hatashita et al., 2014; Nordberg et al., 2013) (Table 2). Because of this, mean levels of amyloid accumulation may not be the most reliable discriminator in MCI. In line, MCI patients with elevated binding of [^11^C]PiB were more likely to convert to AD than prodromal patients who were classified as ‘amyloid negative’ (Okello, Koivunen, et al., 2009).

**Table 2:** Studies examining *in vivo* regional brain uptake using amyloid tracers in studies including amyloid positive MCI

Study	Tau tracer	Study population	Main findings
Okello, Koivunen et al. (2009)	[^11^C]PiB	31 MCI patients (17 amyloid positive and 14 amyloid negative), 26 healthy controls	Compared to healthy controls, elevated cortical tracer retention was found in the frontal, parietal, temporal, occipital, posterior, and anterior cingulate cortex in MCI patients. Then, 14 of 17 (82%) of amyloid positive MCI patients converted to AD, while only 1 out of 14 of amyloid negative cases converted. Converters displayed higher cortical binding in anterior cingulate and frontal areas.
Hatashita et al. (2014)	[^11^C]PiB [^18^F]flutemetamol	36 AD patients, 68 MCI patients, 41 healthy controls	Compared to healthy controls, increased uptake for both tracers was noted in the lateral temporal cortex, anterior cingulate gyrus, frontal cortex, occipital cortex, posterior cingulate gyrus, precuneus, parietal cortex, and sensorimotor cortex in AD. Then, 35 out of 36 AD patients (97.2%) were rated as amyloid positive; 29 out of 68 MCI patients (42.6%) were rated as amyloid positive. No differences were found in MCI patients versus healthy controls. This could be due to the evidenced bimodal SUVR distribution of MCI patients.

Abbreviations: AD, Alzheimer's disease; MCI, mild cognitive impairment; SUVR, standardised uptake value ratio.

Noted strengths of [^11^C]PiB include high amyloid selectivity and affinity, but technical limitations hinder its clinical use in centres without in‐house cyclotrons such as its short decay half‐life (Yeo, Waddell, Khan, & Pal, 2015). Additionally, there are significant operational costs associated with the construction and maintenance of a cyclotron on‐site (Chuck et al., 2005). Data also question the sensitivity of [^11^C]PiB for the detection of early amyloid pathology when compared to cerebrospinal fluid (CSF) Aβ_42_ biomarkers. Specifically, it was found on the trajectory of normal ageing that decreased levels of CSF Aβ_42_ are present before the manifestation of elevated PiB binding (Morris et al., 2010).

### 2.2 ^18^F‐labelled amyloid tracers

To overcome the inherent impracticalities of [^11^C]PiB, three novel ^18^F‐labelled amyloid tracers have been developed: [^18^F]florbetapir (Amyvid), [^18^F]florbetaben (Neuraceq), and [^18^F]flutemetamol (Vizamyl) (Table 1). [^18^F]flutemetamol has a similar neurochemical composition to [^11^C]PiB and is actually a derivative of it (Nelissen et al., 2009), whereas [^18^F]florbetapir and [^18^F]florbetaben are based on the organic compound stilbene (Hatashita et al., 2014). These tracers display a substantially longer half‐life than that of [^11^C]PiB, specifically 110 compared to 20 min. This factor in particular removes the main barrier faced by imaging facilities in pursuing *in vivo* amyloid quantification for clinical use (Jovalekic, Bullich, Catafau, & de Santi, 2016). Moreover, in 2011, the Food and Drug Administration approved use of [^18^F]florbetapir to aid in the diagnostic process in AD (Yang, Rieves, & Ganley, 2012).

**Figure 1:**
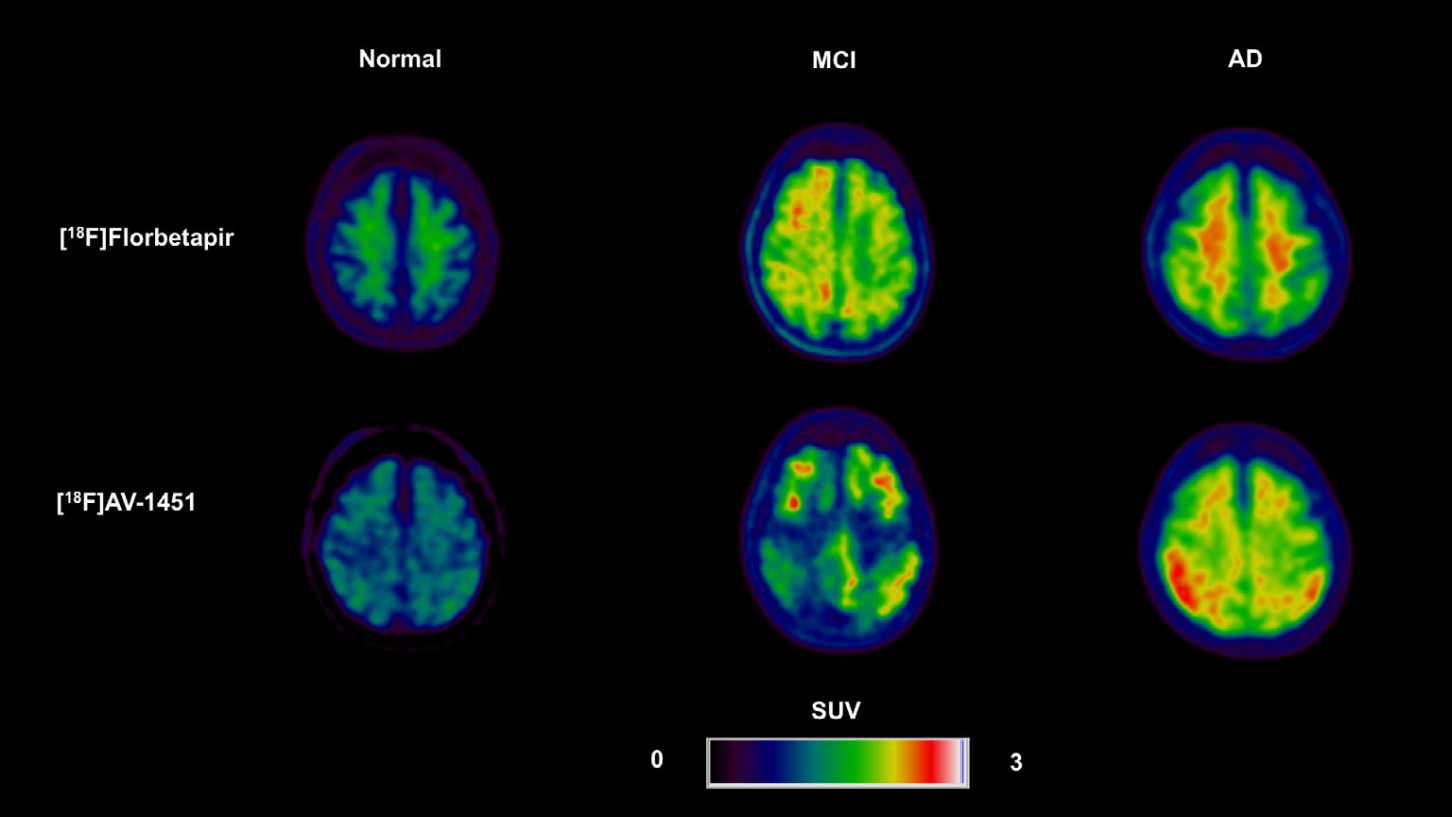
Positron emission tomography (PET) imaging as quantified by standardised uptake values (SUVs) depicting increased tracer uptake for [^18^F]florbetapir and [^18^F]AV‐1451 in an Alzheimer's disease (AD) patient and a mild cognitive impairment (MCI) patient compared to a healthy control [Color figure can be viewed at http://wileyonlinelibrary.com]

In line with its suggested clinical utility, the most widely investigated ^18^F‐labelled amyloid tracer is [^18^F]florbetapir (Figure 1). It was shown to be comparable to [^11^C]PiB, with a high degree of correlation between the two in terms of cortical binding (Landau et al., 2014; Wolk et al., 2012). In a group of individuals with a life expectancy of no more than 6 months, [^18^F]florbetapir showed a sensitivity and specificity of 92 and 100%, respectively, in detecting a significant degree of amyloid plaques. Moreover, the accumulation of amyloid detected by *in vivo* PET imaging correlated with amyloid pathology measured from brain tissue post‐mortem (Clark et al., 2012). In AD, frontal, temporal, occipital, parietal, cingulate, and precuneus cortical areas all show increased amyloid retention as measured by this ligand compared to healthy controls. This finding also holds for MCI patients in the posterior cingulate cortex (Camus et al., 2012). A strong discriminative ability of [^18^F]florbetapir for AD and MCI compared to a cognitively normal status is supported by a number of additional studies (Degenhardt et al., 2016; Johnson et al., 2013; Namiki et al., 2015).

MCI patients rated as Aβ positive using this tracer demonstrated greater longitudinal decline in measures of global cognition, cognitive processing speed, and verbal fluency when compared to Aβ negative patients. AD patients rated as Aβ positive showed a similar pattern on measures of global cognition and verbal fluency (Doraiswamy et al., 2014). Moreover, a positive amyloid scan for MCI patients indicates a greater risk of disease progression to AD (Doraiswamy et al., 2012). While ^18^F‐labelled amyloid tracers are equivalent in their degree of diagnostic accuracy (Morris et al., 2016), [^18^F]florbetapir shows particularly fast brain kinetics relative to others (Wong et al., 2010). When considering the clinical implementation of this modality, recent evidence demonstrates its cost‐effectiveness (Hornberger, Bae, Watson, Johnston, & Happich, 2017) and meaningful impact on the diagnostic confidence for AD by physicians (Boccardi et al., 2016).

While not as widely investigated as [^18^F]florbetapir, a similar pattern of amyloid retention is demonstrated throughout the cortex in AD using both [^18^F]florbetaben (Barthel et al., 2011) and [^18^F]flutemetamol (Nelissen et al., 2009). Although it is not a stilbene derivative, it is important to note that [^18^F]flutemetamol has demonstrated dosimetry comparable with other ^18^F‐labelled radiopharmaceuticals (Heurling, Leuzy, Zimmer, Lubberink, & Nordberg, 2016). Additional commonalities shared by these two tracers with [^18^F]florbetapir include high rates of sensitivity and specificity in diagnosing AD (Barthel et al., 2011; Hatashita et al., 2014; Rowe et al., 2008; Tiepolt et al., 2013; Vandenberghe et al., 2010; Villemagne et al., 2011) and detecting amyloid pathology later confirmed at post‐mortem (Ikonomovic et al., 2016; Sabri et al., 2015; Thal et al., 2015), comparable diagnostic utility to [^11^C]PiB (Lowe et al., 2017; Villemagne et al., 2012), effectiveness in the prediction of conversion from MCI to AD (Ong et al., 2015; Wolk et al., 2018), and the clinical benefit of increased dementia diagnostic confidence (Schipke et al., 2012; Zwan et al., 2017). Associations are shown between Aβ uptake and cognitive dysfunction in AD (Barthel et al., 2011) and MCI (Bahar‐Fuchs et al., 2013) for [^18^F]florbetaben. However, there is a still a strong need to further validate [^18^F]flutemetamol using standardised neuropsychological assessments. Additionally, research suggests that there is a diagnostic advantage of combining structural MRI and PET imaging with this ligand in AD and MCI (Thurfjell et al., 2012).

There is some disagreement between studies regarding the measurement of Aβ deposition using *in vivo* PET. This is due to a range of factors including variability in scanning time, methodology employed during analyses, identified reference regions and regions of interest, attenuation correction, partial volume correction, machines used to scan, and tracer‐specific properties. This has led to some uncertainty regarding a consistent definition of abnormal amyloid levels characteristic of AD (Klunk et al., 2015). In support, Villeneuve et al. found that routinely established thresholds for Aβ positivity using [^11^C]PiB were insensitive and likely to lead to false negatives (Villeneuve et al., 2015). There has been an attempt to solve this problem through the creation of a standardised outcome measure across amyloid‐PET modalities measured in units termed ‘Centiloids’ ranging from 0 to 100 (Klunk et al., 2015). However, there is still a great deal of variability present in the amyloid positivity thresholds derived from this approach (Su et al., 2018). A further limitation of amyloid PET imaging modalities is that there is a lack of concordance with Aβ plaque phases as determined by pathohistological methodology, such as Thal stages (Thal, Rüb, Orantes, & Braak, 2002). For example, [^18^F]flutemetamol PET was unable to accurate predict the target Aβ stages in 27.84% of cases in an end‐of‐life cohort (Thal et al., 2018). Additionally, an amyloid positivity threshold of a standardised uptake value ratio of 1.5 was not sensitive to MCI patients in Thal Phase 1 or 2, and just lowering this value by 0.2 led to approximately 75% of healthy control subjects being classified as having significant brain amyloidosis (Ismail et al., 2019).

## 3 TAU STUDIES

Advances in the measurement of tau pathology in humans using *in vivo* PET radioligands are scarce, but recent, when compared to their amyloid‐specific counterparts. For years, the gold standard in quantifying tau‐derived pathology was either histopathological analysis from post‐mortem tissue or the invasive collection of tau and phosphorylated tau from the CSF by means of lumbar puncture (Saint‐Aubert et al., 2017). This delay in applying a tau ligand was due to several major challenges that were inherently present in the imaging of tau. Unlike amyloid, tau is intracellular, so potential ligands need to be able to cross both the blood–brain barrier and the plasma cell membrane of the neurone. In comparison to amyloid pathology, tau aggregates are present in lower concentrations throughout the brain; therefore, ligands are required to have higher specificity for tau. Additionally, tau has multiple protein conformations and isoforms, which could potentially adversely impact sites for ligand binding (Bischof, Endepols, van Eimeren, & Drzezga, 2017).

The first breakthrough in tau imaging using PET was made using the radioligand [^18^F]FDDNP. However, this methodology concomitantly assesses amyloid plaque burden and cannot be considered a truly selective for tau pathology (Shoghi‐Jadid et al., 2002), and was later shown to have poor binding affinity for NFTs (Thompson et al., 2009). Since then, four families of tracers in particular have shown promise in the assessment of AD and MCI: [^11^C]PBB3, from the same tracer family as [^11^C]PiB, the THK aryquinoline derivatives, the pyrido‐indole derivative [^18^F]AV‐1451, and recently developed second‐generation tau tracers (Table 3).

**Table 3:** Studies examining *in vivo* regional brain uptake using tau tracers in AD and MCI

Study	Tau tracer	Study population	Main findings
Maruyama et al. (2013)	[^11^C]PBB3	Three AD patients, three cognitively normal subjects	Medial temporal regions, including the hippocampus, demonstrated consistently greater [^11^C]PBB3 retention in AD when compared to controls.
Shimada et al. (2017)	[^11^C]PBB3	17 AD patients, 9 MCI patients, 28 healthy controls	Notable differences in tracer uptake were observed in neocortical areas, and particularly the medial temporal cortex for those on the spectrum of AD compared to healthy controls. Medial temporal atrophy on MRI was also observed for this group. Moreover, for those along the spectrum of AD, uptake in frontal and temporo‐parietal junctions were negatively associated with cognitive status, uptake in limbic, paralimbic, and frontoparietal areas were positively associated with dementia status, and uptake in frontal regions was positively associated with frontal executive dysfunction.
Chiotis et al. (2018)	[^11^C]PBB3 [^11^C]THK5351	Four AD patients, five MCI patients	In a mixed group of AD and MCI patients, [^11^C]THK5351 showed greater uptake in medial versus lateral temporal lobe, whereas the inverse was shown for [^11^C]PBB3. [^11^C]PBB3 correlated with PET amyloid uptake. [^11^C]THK5351 and [^11^C]PBB3 regional uptake was negative correlated with cognitive performance.
Chiotis et al. (2016)	[^18^F]THK5317	9 AD patients, 13 MCI patients, 9 healthy controls	Both MCI and AD patients showed increased tau binding in inferior temporal, lateral temporal, lateral occipital, inferior parietal, anterior frontal, lateral occipital cortex, in addition to the precuneus compared to healthy controls. Negative correlations between tau retention and FDG uptake were observed in the frontal cortex, while areas of the neocortex showed positive correlations between tau and amyloid binding on PET.
Lockhart et al. (2016)	[^18^F]THK5351	10 AD patients, 6 healthy controls	Regions including eroded white matter, fusiform gyrus, inferior temporal cortex, lingual gyrus, middle temporal gyrus, occipital cortex, parietal cortex, posterior cingulate, and precuneus demonstrated higher tracer uptake for AD patients relative to healthy controls.
Saint‐Aubert et al. (2016)	[^18^F]THK5317	9 AD patients, 11 MCI patients	[^18^F]THK5317 binding in the parahippocampal, fusiform, inferior, middle and superior temporal gyri, in addition to occipital areas, the middle frontal and posterior cingulate gyri, the parietal operculum, and precuneus were negatively related to cognition for patients along the AD continuum. Outcomes on FDG–PET showed a similar relationship with tau binding and mediated the effects of *in vivo* tau binding on cognition.
Chen et al. (2018)	[^18^F]THK5351 [^18^F]AV‐1451	[^18^F]THK5351: 8 AD patients, 9 healthy controls [^18^F]AV‐1451:12 AD patients, 12 healthy controls	Relative to healthy controls, [^18^F]THK5351 uptake was increased in temporal and occipital cortical areas whereas [^18^F]AV‐1451 uptake was elevated in the temporal cortex for AD patients.
Passamonti et al. (2017)	[^18^F]AV‐1451	15 AD and MCI patients, 13 healthy controls	Compared to healthy controls, those on the AD spectrum exhibited increased binding in all four lobes of the cortex and in the hippocampus.
Wang et al. (2016)	[^18^F]AV‐1451	59 AD patients and healthy controls	Tracer retention was increased in hippocampal and widespread cortical regions in AD patients when compared to controls.
Cho et al. (2016)	[^18^F]AV‐1451	20 AD patients, 15 MCI patients, 20 healthy controls	Relative to controls, the majority of cortical regions assessed demonstrated higher tau uptake in AD. This pattern held for the entorhinal cortex in MCI.
Johnson et al. (2016)	[^18^F]AV‐1451	19 AD and MCI patients, 57 cognitively normal subjects	In a combined patient group, those with AD and MCI showed increased cortical retention of [^18^F]AV‐1451 in temporo‐parietal, parieto‐occipital, precuneus posterior cingulate, and frontal regions compared to controls. Moreover, differences were shown in entorhinal, parahippocampal, inferior temporal, and fusiform cortex. In the patient group, increased inferior temporal uptake was associated with cognitive impairment and dementia severity.
Pontecorvo et al. (2017)	[^18^F]AV‐1451	48 AD patients, 95 MCI patients, 58 older cognitively normal subjects	In frontal, occipital, parietal, and temporal cortex, in addition to the amygdala, anterior hippocampus, anterior and posterior parahippocampus, and fusiform areas AD and MCI patients showed elevated levels of tau binding versus controls.
Whitwell et al. (2018)	[^18^F]AV‐1451	39 typical AD patients	Patients with typical AD demonstrated variability in entorhinal and neocortical tau binding; however, in a cluster analysis comparing high and low uptake groups, those with high entorhinal and neocortical tracer retention showed greatest memory impairment, whilst those with low entorhinal and high neocortical binding showed greatest impairment in other neuropsychological domains.
Murugan et al. (2019)	[^18^F]THK5317	Five AD patients	AD patients demonstrated [^18^F]THK5317 binding in the basal ganglia and thalamus, in addition to the midbrain. Tracer retention was also shown in the isocortical temporal lobe and areas in the lateral parietal and frontal lobes.
Lohith et al. (2019)	[^18^F]MK‐6240	Four AD patients, two MCI patients, four healthy controls	Higher tracer uptake was generally demonstrated in primarily medial temporal lobe areas that included the amygdala, hippocampus, and parahippocampal gyrus in AD/MCI subjects. In two AD subjects in advanced disease stages, uptake was also found in neocortical temporal, frontal, and parietal areas.
Kroth et al. (2019)	[^18^F]PI‐2620	Four AD patients, two healthy controls	Three AD subjects showed asymmetric distributions of tracer retention in temporal regions, the precuneus, and post‐cingulate. One AD subject who was in a very mild stage of the disease demonstrated no uptake.
Wong et al. (2018)	[^18^F]RO‐948	11 AD patients, 4 older controls	AD patients had increased tracer binding relative to older controls in the right hippocampus, entorhinal area, parahippocampus, left middle frontal lobe, fusiform gyrus, middle temporal cortex, inferior temporal lobe, and right inferior parietal lobe.
Bohórquez et al. (2019)	[^18^F]GTP1	65 patients on the spectrum of AD (ranging from prodromal to moderate stages), 15 cognitively normal individuals	Compared to CN individuals, in all AD patients, Braak Stage I/II brain regions demonstrated higher tracer uptake, and in mild and moderate patients on the AD spectrum Braak Stage V/VI regions had elevated tracer retention.

Abbreviations: AD, Alzheimer's disease; FDG, [^18^F]fludeoxyglucose; MCI, mild cognitive impairment; MRI, magnetic resonance imaging; PET, positron emission tomography.

### 3.1 [^11^C]PBB3 and THK tracers

Maruyama et al. validated the use of [^11^C]PBB3, a pyridinated benzothiazole, in providing high‐contrast imaging of tau inclusions in both animals and AD patients. Using a limited sample of three AD patients and three healthy controls, higher tracer accumulation was reported in medial temporal brain areas of AD patients compared to controls. This included the hippocampus (Maruyama et al., 2013). *In vivo* tau burden as detected by this ligand is associated with grey matter atrophy and cognitive impairment (Shimada et al., 2017).

Research suggests that radiotracers from the THK family bind to a different site than that of [^11^C]PBB3 (Lemoine et al., 2017). The latter may be more selective to tau aggregations that have a spatial relationship with amyloid deposits, while the former may show preference for tau closely linked to brain atrophy, CSF tau, and neuropsychological functioning (Chiotis et al., 2018). The first generation of THK radioligands included [^18^F]THK523, [^18^F]THK5105, and [^18^F]THK5117, but demonstrated several flaws that hindered their clinical utility including poor disease discriminability and high white matter retention. Having overcome these limitations, [^18^F]THK5317 and [^18^F]THK5351 may be particularly useful in clinical practice (Saint‐Aubert et al., 2017). [^18^F]THK5317 shows a good ability to differentiate between AD and MCI patients and healthy controls with a pattern of regional uptake that includes selected areas in the temporal, frontal, occipital, and parietal cortex, in addition to the precuneus (Chiotis et al., 2016). Similar results are shown with [^18^F]THK5351 with particularly high retention in temporal brain areas (Harada et al., 2016; Lockhart et al., 2016). A direct comparison of these two tracers revealed that [^18^F]THK5351 has a superior pharmacokinetic profile and greater dissociation from white matter and cerebellar grey matter (Betthauser et al., 2017). However, regional [^18^F]THK5317 binding has shown an inverse relationship with measures of performance on global cognition and episodic memory (Saint‐Aubert et al., 2016). One should interpret results from THK tracers with caution, given evidence that they bind to monoamine oxidase (MAO). For example, in AD, cortical [^18^F]THK5351 binding was associated with MAO‐B measured in post‐mortem brain tissue. Moreover, an MAO‐B inhibitor was shown to actively block THK5351 binding *in vivo* (Harada et al., 2018). Results from another study indicated the detection of suspected off‐target MAO‐B binding for [^18^F]THK5317 in a study of five AD patients (Murugan et al., 2019).

### 3.2 [^18^F]AV‐1451

Compared to [^18^F]THK5317, [^18^F]AV‐1451 (flortaucipir) which is also referred to as [^18^F]T807, has a clearer pattern of cortical uptake in AD patients and demonstrates a lower degree of off‐target binding (Jang et al., 2018) (Figure 1). However, larger effect sizes for cerebellar uptake were noted relative to [^18^F]THK5351 (Chen et al., 2018). Binding of this radioligand mirror the distribution of NFT pathology across the Braak stages (Marquié et al., 2017), and was reduced in areas characterised by Aβ neuritic plaques (Lowe et al., 2016). One study found a classification rate of 85.7% for [^18^F]AV‐1451 in making an accurate clinical diagnosis, including AD and MCI (Passamonti et al., 2017). This tracer also demonstrates high sensitivity and specificity in discriminating AD patients and healthy controls (Maass et al., 2017; Wang et al., 2016). In AD, a similar pattern of cortical uptake is found compared to other tau‐based ligands, whereas patients with MCI demonstrate a more localised pattern of tau deposition in entorhinal regions (Cho et al., 2016; Johnson et al., 2016). While a strength of this radioligand is its specificity for NFT pathology, limitations that pertain to off‐target binding should be acknowledged. A autoradiographic evaluation of [^18^F]AV‐1451 demonstrated that sites exhibiting off‐target binding included the meninges, scalp, basal ganglia, choroid plexus calcifications, blood vessels and potentially red blood cells, areas characteristic of iron deposition, and leptomeningeal melanin (Lowe et al., 2016).

Strong evidence implicates tau pathology as measured by [^18^F]AV‐1451 in the manifestation of dementia‐related cognitive impairment. For example, Pontecorvo et al. show that in a mixed group of Aβ positive subjects, increased tau ligand binding was correlated with global deteriorations in cognition as assessed by the Mini Mental Status Exam (MMSE) and Alzheimer's Disease Assessment Scale‐Cognitive Subscale (ADAS‐Cog) (Pontecorvo et al., 2017). Increased regional tau uptake has also shown associations with impaired domain‐specific neuropsychological performance including memory, language, and visuospatial abilities in numerous variants of AD (Ossenkoppele et al., 2016). Interestingly, patients with a high degree of retention in the entorhinal cortex displayed particularly poor memory functioning (Whitwell et al., 2018). A recent study also links tau burden quantified by [^18^F]AV‐1451 to neurodegeneration, specifically longitudinal brain atrophy (Das et al., 2018). Other neurodegenerative pathology shows unique profiles of tau aggregation as detected by this tracer. For example, patients diagnosed with corticobasal syndrome show retention increases in frontal and parietal cortices compared to those with MCI due to AD (Niccolini et al., 2018). Considering atypical manifestations of AD, those with posterior cortical atrophy display more cortical uptake in occipital areas relative to logopenic progressive aphasia (lvPPA) patients, whilst lvPPA is characterised by more temporal and frontal uptake (Tetzloff et al., 2018). Despite having a favourable pharmacokinetic profile and specificity for tau, further study is required in determining the clinical utility of *in vivo* imaging using [^18^F]AV‐1451 in AD and MCI (Chien et al., 2014; Zhang et al., 2012).

### 3.3 Second‐generation tau tracers

There is a fast pace of development in the tau PET imaging literature with several novel second‐generation tracers currently in development and undergoing validation (Bischof et al., 2017). Evidence indicates numerous advantages attributed to some of these emergent tau tracers, including having a characteristically low relative affinity for MAO‐B in silico (Murugan et al., 2019). One promising second‐generation tau tracer is [^18^F]MK‐6240. A high binding affinity was shown to tau deposits specifically in the brain tissue of AD patients, but not to those in the tissue of patients with other tauopathies or TDP‐43, Aβ, and α‐synuclein. However, off‐target binding to neuromelanin was indicated (Aguero et al., 2019). Cortical tau binding for this tracer was two to three times higher in AD subjects compared to healthy controls (Lohith et al., 2019). [^18^F]PI‐2620 is another recently developed ligand that shows patterns of tau binding in parietal and temporal lobe, in addition to the precuneus and posterior cingulate in AD. A notable lack of off‐target binding, which was an issue faced by tau tracers from prior generations, was also demonstrated (Kroth et al., 2019; Stephens et al., 2018). [^18^F]JNJ311 and [^18^F]JNJ‐067 are structurally different from existing tau tracers and published *in vivo* data on this JNJ family of radioligands is currently unavailable (Leuzy et al., 2019). Wong et al. evaluated three potential tau tracers in amyloid positive AD patients and controls: [^11^C]RO‐963, [^11^C]RO‐643, and [^18^F]RO‐948. Among the three, only [^18^F]RO‐948 was recommended for further investigation as minimal binding was found in controls and patients with AD had region‐specific patterns of tracer uptake consistent with pathohistological data for the spread of tau pathology (Wong et al., 2018). Not only does [^18^F]GTP1 retention correspond to established patterns of brain tau distribution in AD, but this new second‐generation tau tracer was also correlated with performance on cognitive measures, particularly in the earliest stages of AD (Bohórquez et al., 2019; Teng et al., 2018; Teng et al., 2019).

## 4 NEUROINFLAMMATION/TRANSLOCATOR PROTEIN STUDIES

It has been known for some time from the results of histopathological studies that activated microglia localise to Aβ plaques (Perlmutter, Barron, & Chui, 1990) and NFTs (Sheffield, Marquis, & Berman, 2000). Moreover, the results of a genome‐wide association study has implicated the triggering receptor expressed on myeloid cells 2 (*TREM2*) gene, which encodes a microglial cell surface protein, as the second most significant risk factor for late onset AD after *APOE* ε4 (Guerreiro et al., 2013). This has stimulated much research into elucidating the role of inflammation in the pathogenesis of AD.

Advances in the *in vivo* quantification of neuroinflammation have been made through the investigation of translocator protein 18 kDa (TSPO) as a PET imaging target. Throughout the CNS, TSPO is present in glial and ependymal cells and is located on the outer membrane of mitochondria. It is posited that TSPO is involved in a range of cell‐based functions, including cholesterol transport and hormone synthesis (Papadopoulos et al., 2006). However, its exact role and functional significance in relation to the brain immune response is not fully understood. Under normal physiological conditions, there is a low expression of TSPO limited to glial cells. Nevertheless, during neuronal injury or insult, when microglia are activated, TSPO levels in turn experience a significant upregulation (Rupprecht et al., 2010). Moreover, immunohistochemistry studies have confirmed that TSPO upregulation and microglia activation co‐localise spatially following a neurotoxic intervention, suggesting that TSPO can measure neuroinflammation through the detection of activated microglia (Kuhlmann & Guilarte, 2000). Beyond microglia, increased TSPO expression is also observed in reactive astrocytes (Rupprecht et al., 2010). While numerous TSPO radioligands have been developed so far, we will focus on those investigated in AD/MCI patient populations.

### 4.1 [^11^C]PK11195

The most thoroughly researched TSPO radiotracer is [^11^C]PK11195 (Table 4). In a pioneering study, Cagnin et al. demonstrated that patients with AD had a signature pattern of [^11^C]PK11195 uptake in brain areas that included the cingulate, temporoparietal, and entorhinal cortex (Cagnin et al., 2001). Since then, results from this tracer have been mixed and somewhat contradictory. Minimal or small clusters of increased binding in MCI and mild to moderate AD was reported in two studies (Schuitemaker et al., 2013; Wiley et al., 2009). These authors speculated that either microglial activation is implicated later in the disease course or [^11^C]PK11195 is not a sensitive marker of such activity. However, these claims are challenged by findings from studies that used region of interest‐based analyses to show widespread tracer uptake throughout the cortex for AD patients (Edison et al., 2008; Fan, Aman, et al., 2015; Passamonti et al., 2018), particularly in parietotemporal areas (Yokokura et al., 2011) and for MCI patients especially in the frontal cortex (Fan, Aman, et al., 2015; Okello, Edison, et al., 2009). The neurodegenerative effects of this potentially prolonged immune response are hinted at when considering that increased tracer retention has been linked with hippocampal atrophy (Femminella et al., 2016). Utilising [^11^C]PK11195, longitudinal research suggests an initial reduction of microglial activation in prodromal disease stages (Fan et al., 2017) contrasting with a subsequent increase in microglial activation in AD during disease progression (Fan, Okello, et al., 2015).

**Table 4:** Studies examining *in vivo* regional brain uptake using TSPO tracers in AD and MCI

Study	TSPO tracer	Study population	Main findings
Cagnin et al. (2001)	[^11^C]PK11195	8 AD patients, 15 healthy controls	Elevated levels of tracer level were observed in brain areas including the fusiform gyri, left parahippocampal gyrus, left posterior cingulate, inferior and middle temporal gyri, left amygdala, inferior parietal lobules, and to a lesser degree putamen and right pallidum in AD patients when compared to controls. In particular, uptake in the left inferior temporal lobe differentiated AD patients with a sensitivity of 75%.
Schuitemaker et al. (2013)	[^11^C]PK11195	19 AD patients, 10 MCI patients, 21 healthy controls	The only brain region assessed that showed any difference in [^11^C]PK11195 binding between AD patients and controls was the bilateral occipital cortex, where patients showed more binding. No such differences were found when comparing MCI patients to controls.
Wiley et al. (2009)	[^11^C]PK11195	Six patients with AD, six patients MCI patients, five healthy controls	No difference in TSPO binding was found when comparing groups on the AD spectrum with controls in any brain region.
Edison et al. (2008)	[^11^C]PK11195	13 AD patients, 10 healthy controls	Relative to healthy controls, areas in frontal temporal, parietal, and occipital association cortex, in addition to the cingulate and striatum, showed increased tracer uptake in AD patients. Inverse correlations between uptake in posterior cingulate, parietal, and frontal cortical areas and global cognition were found.
Passamonti et al. (2018)	[^11^C]PK11195	16 AD and MCI patients, 13 healthy controls	In a combined group of AD and MCI patients, increased binding was found in brain areas within the occipital, parietal, and temporal cortex, in addition to medial temporal regions including the hippocampus and amygdala. Binding in the precuneus was negatively associated with performance on a measure of delayed recall.
Yokokura et al. (2011)	[^11^C]PK11195	11 AD patients, 10 healthy controls	Medial frontal, parietal, and left temporal cortical areas demonstrated higher [^11^C]PK11195 retention for AD patients compared to controls. Additionally, uptake in the left anterior cingulate, left precuneus, left hippocampus, and left medial frontal cortex showed negative association with global cognitive performance. A similar inverse relationship was found for regional TSPO binding in the posterior cingulate cortex and amyloid uptake on [^11^]C‐PiB in this region.
Okello, Edison et al. (2009)	[^11^C]PK11195	14 MCI subjects, 10 healthy controls	Frontal cortical regions showed increased TSPO binding for MCI patients when compared to controls.
Fan, Brooks, Okello, and Edison (2017)	[^11^C]PK11195	8 AD patients, 8 MCI patients, 14 healthy controls	After a period of 14 months, MCI patients showed reductions in [^11^C]PK11195 in areas including temporal, occipital, parietal, cingulate cortex, and the hippocampus. AD patients showed an increase of approximately 36% in microglial activation relative to controls over this same period of time.
Femminella et al. (2016)	[^11^C]PK11195	Eight AD patients, eight healthy controls	In AD, [^11^C]PK11195 uptake in medial temporal regions and the hippocampus was negatively associated with hippocampal volume as measured by MRI.
Fan, Aman et al. (2015)	[^11^C]PK11195	10 AD patients 10 MCI patients, 16 healthy controls	Cortical retention of [^11^C]PK11195 in areas including the occipital lobe, temporal lobe, hippocampus, parahippocampus, temporal, and precentral and postcentral gyrus was 36–52% higher in AD patients compared to controls. Regions including the temporal, frontal, orbital, straight, parietal gyrus, insula, putamen, and occipital lobe were 28–36% higher in MCI relative to controls. Throughout the four lobes of the cortex, and the insula, thalamus, and hippocampus microglial activation and amyloid load measured by [^11^C]PiB were positively correlated, a similar but not as widespread relationship was evidenced in MCI. TSPO uptake in frontal, temporoparietal, and occipital cortex was negatively correlated with global cognition, while regional associations were also found with cerebral glucose hypometabolism on FDG PET.
Yokokura et al. (2017)	[^11^C]PK11195 [^11^C]DPA‐713	[^11^C]‐PK11195: 10 AD patients, 10 healthy elderly controls [^11^C]DPA‐713:7 AD patients, 12 healthy elderly controls	In temporal, occipital, parietal, frontal, cingulate, parahippocampal, in addition to the cerebellum, hippocampus, amygdala, caudate, putamen, and thalamus showed greater [^11^C]DPA‐713 uptake than controls; however when using [^11^C]PK11195 only the precuneus showed this pattern. While regional [^11^C]DPA‐713 uptake demonstrated an inverse relationship with cognition, no such significant relationship was shown using [^11^C]PK11195.
Yasuno et al. (2008)	[^11^C]DAA1106	10 AD patients, 10 healthy controls	[^11^C]DAA‐1106 binding was upregulated in the cerebellum, prefrontal cortex, parietal cortex, temporal cortex, occipital cortex, anterior cingulate cortex, and striatum in AD patients compared to controls.
Kreisl et al. (2013)	[^11^C]PBR28	19 AD patients, 10 MCI patients, 13 healthy controls	[^11^C]PBR28 binding was elevated in prefrontal, inferior parietal, temporal, precuneus, posterior cingulate, occipital, hippocampus, entorhinal cortex in AD patients compared to controls. No such effects were found for MCI patients. Region‐specific binding was positively associated with worse performance on global cognition, dementia severity, memory, visuospatial ability, and executive functioning. A similar relationship was found in AD with tracer uptake and brain atrophy.
Lyoo et al. (2015)	[^11^C]PBR28	25 AD patients, 11 MCI patients, 21 healthy controls	Compared to healthy controls, temporal and parietal brain areas demonstrated significantly higher uptake in AD patients. Relative significant differences for MCI patients were not evidenced.
Hamelin et al. (2016)	[^18^F]DPA‐714	64 AD patients, 32 healthy controls	Patients with AD, including the prodromal form, demonstrated greater tracer uptake in regions that include the precuneus, parietal, temporal cortex, and medium and posterior cingulate compared to controls. This uptake was positively associated with performance on global cognition and grey matter volume. This also holds for regional amyloid uptake.
Suridjan et al. (2015)	[^18^F]FEPPA	21 AD patients, 21 healthy controls	Temporal, frontal, parietal, and occipital cortical regions and the hippocampus demonstrated increased tracer retention for AD patients compared to controls. This also held for the posterior limb of the internal capsule and the cingulum bundle. Regional uptake was associated with impairment in visuospatial ability and language ability.
Varrone et al. (2015)	[^18^F]FEMPA	10 AD patients, 7 healthy controls	Analyses revealed that higher [^18^F]FEMPA uptake was observed in medial temporal, lateral temporal, and posterior cingulate cortex, in addition to regions including the putamen, caudate, thalamus, and cerebellum for AD patients when compared to controls.
Kreisl et al. (2016)	[^11^C]PBR28	14 AD patients, 8 healthy controls	Relative to controls, elevated radiotracer binding was demonstrated in the inferior parietal lobule, occipital cortex, precuneus, entorhinal cortex, hippocampus, inferior, and middle temporal cortex for AD patients. Annual increases in [^11^C]PBR28 binding ranging from 2.5 to 7.7% was also observed in AD. Regional increases in patients were positively correlated with dementia severity, and brain atrophy.
Hamelin et al. (2018)	[^18^F]DPA‐714	Baseline: 52 AD patients, 17 healthy controls Follow‐up: 21 AD patients, 13 healthy controls	At baseline, temporal and parietal brain areas had significantly higher tracer retention in AD patients relative to controls. Tracer retention was positive correlated with a worsening of dementia and cognitive status, in addition to brain atrophy. Annual increases of 13.2% were observed for AD patients in terms of TSPO tracer binding.
Yasuno et al. (2012)	[^11^C]DAA1106	10 AD patients, 7 MCI patients, 10 healthy controls	Compared to controls, MCI patients demonstrated increased TSPO binding in the striatum, lateral temporal, parietal, and anterior cingulate cortex. This pattern held for AD in these regions in addition to the medial prefrontal cortex.
Fan et al. (2018)	[^11^C]PBR28	13 MCI patients, 9 healthy controls	MCI patients had greater tracer uptake in the temporal lobe, post‐cingulate cortex, thalamus, medial temporal lobe, hippocampus, amygdala, and cerebellum when compared to healthy controls.
Knezevic et al. (2017)	[^18^F]FEPPA	11 aMCI patients, 14 healthy controls	Region‐specific TSPO binding was not significantly different between aMCI patients and controls. However, there was a positive correlation between TSPO binding and amyloid binding on [^11^C]PiB in aMCI in the hippocampus.
Parbo et al. (2018)	[^11^C]PK11195	6 AD patients, 20 MCI patients, 20 healthy controls	In frontal, posterior cingulate, parahippocampal, lateral and posterior temporal cortex, precuneus, and hippocampus, increased TSPO binding was found in MCI patients compared to controls. In a mixed group of 16 MCI and AD patients with high Aβ, microglial activation was correlated with PiB amyloid uptake in frontal, parietal, and lateral temporal areas.
Fan, Okello, Brooks, and Edison (2015)	[^11^C]PK11195	8 AD patients, 14 healthy controls	AD patients demonstrated increased microglial tracer uptake in frontal, parietal, occipital, temporal cortical areas, in addition to the striatum and hippocampus relative to controls. Regionally distributed uptake on [^11^C]PK11195 was correlated positively with amyloid binding on [^11^C]PiB, and negatively correlated with brain glucose metabolism on FDG PET.
Parbo et al. (2017)	[^11^C]PK11195	42 MCI patients, 10 healthy controls	In amyloid positive MCI subjects, TSPO binding was elevated in frontal, parietal, and lateral temporal regions compared to controls. Moreover, for this group, a positive correlation on binding outcome was shown between [11C]PK11195 and [^11^C]PiB in frontal, temporal, and parietal brain areas.
Dani et al. (2018)	[^11^C]PBR28	16 AD patients, 16 MCI patients, 19 healthy controls	In AD and MCI patients, brain region‐based clusters of positive correlations were found between neuroinflammation on [^11^C]PBR28 and amyloid retention on [^18^F]flutemetamol. A positive relationship also existed between TSPO binding and tau aggregation measured by [^18^F]AV‐1451.

Abbreviations: AD, Alzheimer's disease; aMCI, amnestic mild cognitive impairment; FDG, [^18^F]fludeoxyglucose; MCI, mild cognitive impairment; MRI, magnetic resonance imaging; PET, positron emission tomography; TSPO, translocator protein.

Results pertaining to the relationship between [^11^C]PK11195 binding and cognitive status are also conflicting. Three studies found inverse correlations between MMSE score and tracer uptake (Edison et al., 2008; Fan, Aman, et al., 2015; Yokokura et al., 2011) and one demonstrates a negative correlation between tracer uptake in the precuneus and episodic memory performance measured by the Rey Auditory Verbal Learning Test (Passamonti et al., 2018), which suggests a role for microglial activation in disease severity. However, others failed to find similar associations between [^11^C]PK11195 retention and neuropsychological performance (Schuitemaker et al., 2013; Yokokura et al., 2017). This pattern of contradictory results could possibly be attributed to small sample sizes, limitations of the tracer and variability in study methodology.

Several technical limitations of [^11^C]PK11195 were found, including its short half‐life of approximately 20 minutes that is a barrier for centres without a costly on‐site cyclotron, low brain uptake, and importantly low signal to noise ratio. It also exhibits a high degree of nonspecific binding (Ching et al., 2012). This nonspecific binding may apply to targets including brain‐based lipids (Hatty et al., 2014) and α1‐acid glycoprotein (Lockhart et al., 2003). Additional clinical translation difficulties are imposed on the molecule through carbon‐11 labelling (Chauveau, Boutin, Van Camp, Dollé, & Tavitian, 2008). However, suboptimal modelling of this tracer is likely its most pressing concern as there has been substantial difficulty in the definition of a true reference region, which is an area absent of binding, for [^11^C]PK11195 (Chauveau et al., 2008). When using an arterial plasma input function, the use of a reference region allows for valid quantification of binding potential (Cunningham, Parker, Rabiner, Gee, & Gunn, 2005). In fact, variability in the findings of studies discussed using [^11^C]PK11195 could, at least in part, reflect the use of various and inconsistent methods for reference region quantification. To account for this issue, an automatic supervised clustering procedure utilising *a priori* kinetic classes has been developed to extract grey matter estimates that can be reliably classified as reference region tissue (Turkheimer et al., 2007).

### 4.2 Second‐generation TSPO tracers

The shortcomings of [^11^C]PK11195 were overcome with the advent of second‐generation TSPO ligands, which notably had an improved signal‐to‐noise ratio and higher binding affinity compared to [^11^C]PK11195 (Edison & Brooks, 2018). While many second‐generation TSPO tracers have been discovered, we will cover only those that have been most widely used in humans and specifically in AD or MCI. These include [^11^C]PBR28, [^18^F]DPA‐714, [^18^F]FEPPA, [^18^F]FEMPA, and [^11^C]DAA1106 (Table 4). Findings from studies examining neuroinflammation, as measured by increased tracer retention, in AD patients using these newer ligands are more consistent than [^11^C]PK11195. Specifically, increased levels of region‐specific TSPO binding in numerous cortical areas are demonstrated in AD patients compared to healthy controls (Hamelin et al., 2016; Kreisl et al., 2013; Lyoo et al., 2015; Suridjan et al., 2015; Varrone et al., 2015; Yasuno et al., 2008). The temporal pattern of neuroinflammation over the course of the AD has also been well‐characterised through longitudinal investigations. In AD patients, yearly average increases in TSPO binding that ranged from 2.5 to 7.7% were shown using [^11^C]PBR28 (Kreisl et al., 2016), whilst for [^18^F]DPA‐714 an elevated annual change of 13.2% in tracer binding was displayed (Hamelin et al., 2018).

A change in role of activated microglia is supported by a large longitudinal study utilising [^18^F]DPA‐714 (Hamelin et al., 2016; Hamelin et al., 2018). Participants with MCI and higher initial TSPO binding had a slower rate of decline measured by the Clinical Dementia Rating and smaller increase in TSPO binding than those with lower initial TSPO binding. These results, coupled with the aforementioned [^11^C]PK11195 studies, have led to the proposal of a dual peak hypothesis of neuroinflammation in AD (Fan et al., 2017). This suggests that the early peak in activated microglia in MCI patients is initially protective, attempting to remove Aβ, whereas the later peak in activated microglia is detrimental. Associations between TSPO expression and clinical outcome for individuals on the spectrum of AD may only be observable for neuroimaging data collected during rising rather than declining phases of these peaks. It is important to note that results from TSPO imaging studies that include MCI patients may reflect either a rising or declining PET signal. While different phenotypes of activated microglia are detectable in pathological studies (Tang & Le, 2016), PET imaging utilising TSPO is unable to differentiate the microglial subtype.

Similar to [^11^C]PK11195, *in vivo* increases in TSPO binding are associated with impairments in global cognition and memory (Hamelin et al., 2018; Kreisl et al., 2013), but also extend to domains that include visuospatial and language ability, and executive functioning, in addition to dementia severity and brain atrophy (Hamelin et al., 2018; Kreisl et al., 2013; Kreisl et al., 2016; Suridjan et al., 2015). However, some studies demonstrate no such correlations with the ADAS‐Cog or MMSE (Yasuno et al., 2008; Yasuno et al., 2012) whilst one reported an opposite pattern, where MMSE scores were positively related to [^18^F]DPA‐714 binding (Hamelin et al., 2016). This latter result could possibly be attributed to the inclusion of prodromal AD patients in the study sample. Overall, the nature of the correlation between inflammatory changes and cognitive performance requires further investigation. When clarifying regional uptake patterns of second‐generation TSPO ligands in MCI, further research is needed as well. This is due to some studies showing striking patterns of upregulated cortical tracer retention when compared to healthy controls, especially in the temporal lobe (Fan et al., 2018; Yasuno et al., 2012), and others showing no such significant differences (Knezevic et al., 2017; Kreisl et al., 2013).

It is imperative to acknowledge the primary limitation of these second‐generation tracers, which is their particular sensitivity to a single nucleotide polymorphism (SNP) of the *TSPO* gene. Genetic variation in the SNP rs6917 results in different patterns of binding affinity to TSPO: high affinity binders, low affinity binders, and mixed affinity binders. Therefore, it is essential to test for these genetic polymorphisms when examining data from these tracers and exclude low affinity binders, which consist of approximately 10% of the population, from analyses (Edison & Brooks, 2018; Yoder et al., 2013). Not being influenced by this TSPO polymorphism can be considered an advantage of [^11^C]PK11195.

## 5 INTERPLAYS BETWEEN TAU, AMYLOID, AND MICROGLIA

Overall, tau, amyloid, and TSPO radiotracers show good ability in the detection of AD and MCI, in addition to associated neuropathology (Table 5). However, the role of prolonged microglial activation in the initiation and exacerbation of amyloid and tau neuropathology in AD remains contested. Pro‐inflammatory cytokines, which are produced by microglia in response to neuronal damage (Smith, Das, Ray, & Banik, 2012), experience a chronic activation due to Aβ exposure. This leads to cellular neurotoxicity and potentially to clinical deterioration in AD (Serrano‐Pozo, Betensky, Frosch, & Hyman, 2016). These cytokines have also been implicated in the manifestation of tau in its pathological state, specifically in tau hyperphosphorylation and NFT generation (Maphis et al., 2015). Parbo et al. posit a model where amyloid pathology precedes and activates microglia. Resultant cytokines can then lead to the promotion of tau pathology (Parbo et al., 2018). In support of this, PET imaging with both [^11^C]PK11195 and second‐generation TSPO ligands have shown positive correlations between regional TSPO binding and amyloid load in AD and MCI (Dani et al., 2018; Fan, Aman, et al., 2015; Fan, Okello, et al., 2015; Hamelin et al., 2016; Knezevic et al., 2017; Parbo et al., 2017; Parbo et al., 2018). Whilst older and using smaller samples, studies indicating negative or no association between microglial activation and Aβ accumulation (Kreisl et al., 2013; Okello, Edison, et al., 2009; Wiley et al., 2009; Yokokura et al., 2011) highlights the need for future research to clarify the nature of this relationship using *in vivo* brain imaging technology.

**Table 5:** Studies examining sensitivity and specificity (in %) of amyloid, tau, and TSPO tracers in the detection of AD and neuropathology

Tracer target	Tracers	Studies	Discriminating AD from healthy controls	Detecting AD‐related neuropathology
Amyloid	[^11^C]PiB [^18^F]florbetapir [^18^F]flutemetamol [^18^F]florbetaben	Rowe et al. (2010), Clark et al. (2012), Camus et al. (2012), Hatashita et al. (2014), Barthel et al. (2011), Rowe et al. (2008), Villemagne et al. (2011), Tiepolt et al. (2013), Vandenberghe et al. (2010), Sabri et al. (2015), Ikonomovic et al. (2016)	Sensitivity: 80–100% Specificity: 66–96%	Sensitivity: 91–97.9% Specificity: 90–100%
Tau	[^18^F]AV‐1451 [^18^F]THK5351	Chen et al. (2018), Wang et al. (2016), Maass et al. (2017)	Sensitivity: 78.8–100% Specificity: 45–95%	Not available
TSPO	[^11^C]PK11195	Cagnin et al. (2001)	Sensitivity: 75%	Not available

Abbreviations: AD, Alzheimer's disease; TSPO, translocator protein.

Two very recent studies have sought to investigate whether associations also exist between TSPO binding and both tau retention on [^18^F]AV‐1451 and amyloid load. The first found no association between the two, but used a sample of patients primarily with prodromal disease (Parbo et al., 2018). The second study evidenced a positive relationship between neuroinflammation and both tau and amyloid pathology in patients with AD and MCI, with similar targeted clusters of cortical regions (Dani et al., 2018). Furthermore, the correlation between tau and neuroinflammation was demonstrated even in participants without significant amyloid burden, suggesting a process independent of Aβ. These results offer the first *in vivo* evidence in AD and MCI patients that neuroinflammation and tau pathology have a pathophysiological link.

## 6 CONCLUSIONS

Molecular imaging with PET has shed light into the complex interplay between Aβ, tau, and neuroinflammation in AD and MCI and helped to clarify to what extent these are part of the normal ageing process or if they represent a distinct pathophysiological process. It has also assessed the patterns of neuropathological regional depositions and their relation to cognitive decline, disease progression and, ultimately, neurodegeneration.

*In vivo* imaging of dementia‐related pathology through the reviewed PET radioligands has several benefits. First, a preclinical detection of the disease can be achieved by examining early molecular changes. In addition, the differential diagnosis process between AD and other neurodegenerative disorders can be refined through a focus on characteristic neuropathological mechanisms. From a research perspective, these techniques allow for the study of relationships between cognition and specific neurodegenerative processes across the AD continuum. Finally, these PET molecular targets can serve as surrogate markers for treatment efficacy and may represent potential means of therapeutic intervention.

This review has demonstrated promising results regarding the role of PET molecular imaging in the diagnosis of AD and MCI and their underlying pathological processes, supporting its use as a research tool and an adjunct in clinical practice. Also highlighted are a number of areas of uncertainty and various tracers' limitations. The interplay between amyloid, tau, and neuroinflammation is an exciting new area of investigation that has only recently become possible through the development of an expanded repertoire of PET tracers. Given the proposal that the role of neuroinflammation in AD pathogenesis changes over the disease course, future multitracer longitudinal studies are required.

### ACKNOWLEGMENTS

Data collection and sharing for this project, specifically generation of figures depicting PET images, was funded by the Alzheimer's Disease Neuroimaging Initiative (ADNI) (National Institutes of Health Grant U01 AG024904) and DOD ADNI (Department of Defense award number W81XWH‐12‐2‐0012). ADNI is funded by the National Institute on Aging, the National Institute of Biomedical Imaging and Bioengineering, and through generous contributions from the following: AbbVie, Alzheimer's Association; Alzheimer's Drug Discovery Foundation; Araclon Biotech; BioClinica, Inc.; Biogen; Bristol‐Myers Squibb Company; CereSpir, Inc.; Cogstate; Eisai, Inc.; Elan Pharmaceuticals, Inc.; Eli Lilly and Company; EuroImmun; F. Hoffmann‐La Roche Ltd. and its affiliated company Genentech, Inc.; Fujirebio; GE Healthcare; IXICO Ltd.; Janssen Alzheimer Immunotherapy Research & Development, LLC.; Johnson & Johnson Pharmaceutical Research & Development LLC.; Lumosity; Lundbeck; Merck & Co., Inc.; Meso Scale Diagnostics, LLC.; NeuroRx Research; Neurotrack Technologies; Novartis Pharmaceuticals Corporation; Pfizer Inc.; Piramal Imaging; Servier; Takeda Pharmaceutical Company; and Transition Therapeutics. The Canadian Institutes of Health Research is providing funds to support ADNI clinical sites in Canada. Private sector contributions are facilitated by the Foundation for the National Institutes of Health (http://www.fnih.org). The grantee organization is the Northern California Institute for Research and Education, and the study is coordinated by the Alzheimer's Therapeutic Research Institute at the University of Southern California. ADNI data are disseminated by the Laboratory for Neuro Imaging at the University of Southern California. ADNI was not involved in the writing of this manuscript or the decision to submit the manuscript for publication. Mr A.C., Dr P.E.V., Dr G.P., Dr O.C., and Dr G.D. report no disclosures. M.P. research is supported by Michael J Fox Foundation for Parkinson's Research, Edmond and Lilly Safra Foundation, CHDI Foundation, Glaxo Wellcome R&D, Life Molecular Imaging, Invicro, Curium, Medical Research Council (UK), AVID Radiopharmaceuticals, National Institute for Health Research, Alzheimer's Research UK, and European Commission IMI2 fund.

## CONFLICT OF INTEREST

The authors do not report any actual or potential conflict of interest relevant to this work.
